# Interest in Genetic Feedback for Alcohol Use Disorder and Related Substance Use and Psychiatric Outcomes among Young Adults

**DOI:** 10.3390/brainsci10121007

**Published:** 2020-12-18

**Authors:** Morgan N. Driver, Sally I-Chun Kuo, Danielle M. Dick, on behalf of the Spit for Science Working Group

**Affiliations:** 1 Department of Human and Molecular Genetics, Virginia Commonwealth University School of Medicine, Richmond, VA 23298, USA; 2 Department of Psychology, Virginia Commonwealth University, Richmond, VA 23284, USA; ickuo@vcu.edu

**Keywords:** alcohol use disorder, genetic feedback, genetic literacy, genetic interest, personalized feedback, psychiatric conditions

## Abstract

An exponential growing number of individuals are accessing genetic risk information via direct to consumer companies. Alcohol dependence is the third most accessed genetic risk score on a publicly available direct to consumer website. Better understanding of the degree to which individuals are interested in receiving personalized genetic feedback, the factors that relate to interest, and genetic knowledge will be critical to lay the foundation for precision medicine initiatives, especially for substance use and psychiatric outcomes, where less is known. To assess interest in receiving genetic feedback for alcohol use disorder (AUD) and understanding of genetic concepts related to psychiatric conditions, we conducted a survey with participants recruited from a registry that enrolled incoming cohorts of freshmen at an urban public university; 205 participants (76.5% female; 58.9% self-reported as White; M_age_ = 24.48 years) completed the survey. Results indicated that participants are highly interested in receiving genetic feedback for AUD (79.0%) but there is a lack of understanding of complex genetic concepts in a sizable proportion of the sample (25.4%). Additional research is needed to assess how to address this lack of knowledge before genetic feedback for AUD can be returned in a way that benefits the individual.

## 1. Introduction

Genome-wide association studies are rapidly increasing our understanding of the complex genetic architecture of alcohol use disorder (AUD), as well as related substance use and psychiatric conditions [1,2,3,4]. One goal of genome-wide association studies (GWAS) is to generate findings that can be used for enhanced clinical prediction in the future [5,6]. Genome-wide polygenic scores for AUD created from GWAS studies index an individual’s overall genetic risk across the genome, which can aid in identifying individuals at-risk for AUD to help improve current prevention strategies and aid in earlier intervention. Presently, genome-wide polygenic scores capture about 2.5–3.5% of the variance for AUD [7]. Despite the low predictive ability of current genome-wide polygenic scores, personalized genetic information is already being provided to increasingly large numbers of the public [8]. Public websites allow individuals to upload raw genetic data obtained from direct-to-consumer genetic tests to compute genetic risk scores for a variety of conditions. User data from one of these popular websites (impute.me) illustrates an exponential increase in accessing genetic risk scores over the last several years with alcohol dependence being the third most accessed genetic risk score out of >1500 conditions [8].

However, there are concerns that individuals may misinterpret or misunderstand their complex genetic results. There is wide variability in genetic literacy across extant studies, ranging from 42.2–83.6% accuracy [9,10,11,12,13,14,15,16]. Studies have examined whether demographic variables, such as gender, education/income, or race/ethnicity, are associated with individuals’ genetic literacy; however, findings have been mixed [10,12,14,15,16]. Concerningly, there is evidence of substantial misunderstanding of complex genetic concepts. A large survey (*N* > 5000) of participants with at least a secondary education, from 78 countries, found that 30% of participants assumed that one individual gene was responsible for causing complex disorders and 25% of participants agreed with the statement that their destiny was written in their genes [10]. Additionally, an Australian-based study found that approximately 25% of respondents misunderstood the role of environmental factors in complex disease [16]; they did not understand that lifestyle choices as well as other environmental factors could reduce the expression of a genetic predisposition. These misinterpretations related to genetic susceptibility to complex conditions are likely to impact how individuals interpret their personalized genetic feedback that suggests they are at increased risk for a condition.

In order to gauge interest in receiving genetic feedback for AUD and to assess individuals’ understanding of the role of genetic and environmental influences in complex psychiatric conditions, we conducted a survey in a sample of participants recruited from a registry that enrolled incoming cohorts of freshman at a diverse urban public university [17]. Young adults are the focus of this study because they are at higher risk for AUD, with rates of AUD peaking in the 20s, as well as entering a high-risk age range for the onset of many psychiatric conditions [18]. College students in particular are at an elevated risk compared to non-college attending peers due to higher rates of alcohol use [19]. Additionally, youth and young adults are high consumers of new technologies, with a majority using various digital technologies to access health information [20]. Therefore, emerging adults may be more likely to access their genetic risk information as well as more likely to benefit from receiving genotypic information for AUD, which uniquely positions emerging adults as the target population for this study. 

The purpose of this study was to gauge individuals’ interest in receiving genetic feedback for AUD and related psychiatric conditions, examine variables associated with interest in genetic feedback, and assess genetic knowledge as it relates to psychiatric conditions in a sample of young adults. We hypothesized that a significant proportion of the sample (~80%) would be interested in receiving genetic feedback for AUD, based on studies of older individuals [21,22]. Additionally, we hypothesized that there would be a sizable proportion (~25%) of participants with poor genetic knowledge related to substance use and mental health conditions based on current literature that shows 25–30% of individuals have misconceptions about the meaning of genetic risk and the genetics of complex diseases [10,16]. Our hypotheses were pre-registered using the Open Science Framework (osf.io/v4eaw). 

## 2. Materials and Methods

### 2.1. Sample and Procedures 

Data for the present study came from the Spit for Science (S4S) Registry, a university-wide research project aimed at understanding how genes and environments impact substance use and mental health outcomes across the college years and beyond [17]. The S4S Registry invites incoming first-time students aged 18 and older to complete an online survey and provide a saliva sample for genotyping. Participants are then invited to complete a follow-up survey each spring while attending college. Data collection for S4S began in the fall of 2011, and five cohorts (cohorts 2011, 2012, 2013, 2014 and 2017) of incoming students have been enrolled (*N* = 12,365), with 68% participation rate. When students picked up their compensation for completing the survey, they were invited to provide a saliva sample for genotyping; 97% of participants provided a saliva sample. 

Analyses of the present study focused on a subset of participants from the S4S Registry who completed an additional online follow-up survey designed to assess genetic knowledge and interest in receiving genetic feedback for substance use and mental health conditions. In June 2020, invitation emails were sent to 1737 eligible participants who met the inclusion criteria: (1) had enrolled in Spit for Science as part of cohort 4 from 2014–2015 and (2) had GWAS data available such that they provided a DNA sample which passed quality control measures. Two hundred twenty-six individuals consented to participate in the survey by October 2020, 205 individuals completed the survey and were included in the analyses and 21 participants were excluded from the present analyses due to incomplete survey data (0–34.9% completion). All surveys in S4S, including this follow-up survey, were administered using the REDCap software [23]. Informed consent was obtained electronically. Participants were compensated with a $5 Amazon gift card after completion of this follow-up survey. All procedures were approved by the University’s Institutional Review Board. 

Of the 205 participants who completed this follow-up survey, 76.5% of the sample was female; 58.9% self-identified as White, 18.3% Asian, 13.4% African American/Black, 3.5% Hispanic/Latino, 5.4% more than one race, 0.5% Native Hawaiian/Other Pacific Islander; and the mean age of the sample was 24.48 years (SD = 0.36). To determine whether this subset of individuals who completed the follow-up was different from the full sample of invited participants, a series of comparison tests were run on demographic and other relevant variables. No differences between the full sample of invited participants and this subset were detected on age or anxiety and depressive symptoms, alcohol problems, or nicotine use at enrollment in S4S. However, the subsample was more likely to be female (*X^2^ =* 17.566, *df* = 1, *p* < 0.001) and White (*X^2^ =* 3.871, *df* = 1, *p* = 0.049). The subsample was also less likely to have used cannabis as a freshman in college (*X^2^ =* 5.0183, *df* = 1, *p* = 0.025) and more likely to have a positive family history of depression/anxiety (*X^2^ =* 4.448, *df* = 1, *p* = 0.035); no differences for family history of alcohol problems or family history of drug problems were detected. 

### 2.2. Measures

A copy of all survey items used in the study is available in the Supplementary Materials.

#### 2.2.1. Interest in Receiving Genetic Feedback 

Interest in receiving genetic feedback for AUD, as well as other related substance use and mental health conditions, including nicotine use disorder (NUD), cannabis use disorder (CUD), depression, and anxiety, was assessed with the item “Would you be interested in receiving personalized genetic feedback for any of the following mental health conditions?” in which participants were instructed to select all options that applied. 

#### 2.2.2. Genetic Knowledge 

Genetic knowledge was measured using an adapted version of the Public Understanding and Attitudes towards Genetics and Genomics (PUGGS) Questionnaire Section 3 [24]. The measure consists of 11 items that assess individuals’ understanding of the genetic and environmental contributions to psychiatric conditions. Items are rated with response options of “true”, “false”, and “don’t know”. An example item is: “A person’s substance use disorder is influenced by many different genes.” Section 3 of the PUGGS questionnaire is a valid measure to test young adults’ understanding of complex genetic concepts, with a Cronbach’s alpha of 0.67 [24]. Overall genetic knowledge scores were calculated as the sum of correct answers for the 11 genetic knowledge items. Correct responses were scored as 1 and incorrect and don’t know responses were scored as 0. Pro-rating was used to account for missing items for individuals with >50% of the genetic knowledge items. 

#### 2.2.3. Demographic Variables 

Sociodemographic variables included sex (female = 1; male = 0), self-reported race/ethnicity (White, Black/African American, Asian, Hispanic/Latino, Native Hawaiian/Other Pacific Islander, American Indian/Alaskan Native and more than one race), and age. Due to the majority of participants self-identifying as White (58.9%), self-report race/ethnicity was coded as a binary variable (0 = White; 1 = racial/ethnic minority) in the analyses. 

#### 2.2.4. Substance Use

Alcohol use and cannabis use were measured using items from the Alcohol Use Disorders Identification Test (AUDIT) [25] and Cannabis Use Disorders Identification Test (CUDIT) [26], respectively, which assess frequency of use. Response options for frequency of alcohol use include “never”, “monthly or less”, “2 to 4 times a month”, “2 to 3 times a week”, and “4 or more times a week”, and the variable was coded with a range of 0–4. Nicotine use was measured using an adapted item from the National Survey on Drug Use and Health [27] that assesses past 30-day use. In view of the distributions for nicotine use and cannabis use showing that most participants were non-users (80.5% and 59.6%, respectively), we coded nicotine use and cannabis use into a binary variable to index user versus non-user.

#### 2.2.5. Anxiety and Depressive Symptoms 

Abbreviated scales from the Symptom Checklist-90 were used to assess anxiety and depressive symptoms occurring within the last 30 days [28]. Four items measured anxiety symptoms, and four items assessed depressive symptoms. Responses were rated on a 5-point Likert-type scale (“not at all”, “a little bit”, “moderately”, “quite a bit”, and “extremely”). Anxiety symptoms were calculated as the sum of four SCL-90 anxiety items. Depressive symptoms were calculated as the sum of four SCL-90 depression items. Pro-rating was used to account for missing items for individuals with >50% of the SCL-90 depressive/anxiety symptom items. Higher scores indicate higher levels of symptoms.

#### 2.2.6. Family History 

Family history was measured using items from a previous S4S survey [29]. Participants answered separate questions about drinking problems, problems with depression/anxiety, or problems with other drugs for four types of relatives: mother, father, aunts/uncles/grandparents, and siblings. An example question was: “Do you think your biological mother has ever had problems with alcohol (by problems with alcohol we mean that her alcohol use caused problems at home, at work, with her health, or with the police, or that she received alcohol treatment)?”. The questions were repeated for each type of relative. Response options for each question were “yes” and “no”. Family history items related to alcohol problems were combined into an overall binary variable to indicate whether or not the participant has any positive family history of alcohol problems. These methods were used to generate a binary family history item for other drug problems and a binary family history item for depression/anxiety as well.

### 2.3. Analytic Plan 

Descriptive analyses were used to describe demographic information, interest in receiving genetic feedback (frequencies for each condition) and overall genetic literacy scores (median and interquartile range). To examine the relationship between demographic characteristics, substance use and mental health variables, and family history with individuals’ interest in receiving genetic feedback, we conducted a series of *t*-tests and chi-square tests. For quantitative variables, we used *t*-tests to compare means of variables of interest (e.g., age, genetic knowledge) between the group interested in receiving genetic feedback and the group not interested in receiving genetic feedback. For qualitative/categorical variables, we conducted chi-square tests to assess whether there was a relationship between variables of interest (e.g., family history, substance use) and interest in receiving genetic feedback. Tests were run separately for interest in receiving feedback for each of the five outcomes (AUD, NUD, CUD, depression, anxiety). Additionally, we conducted a series of univariate linear regression analyses to examine the relationship between demographic characteristics, substance use and mental health variables, and family history with overall genetic knowledge. Separate linear regressions were run for each of these variables, with overall genetic knowledge as the dependent variable in the regression models. All analyses were conducted using R 3.6.2 software [30].

## 3. Results

### 3.1. Interest in Receiving Genetic Feedback

Table 1 shows the frequencies of interest in receiving genetic feedback for AUD and related psychiatric conditions in the sample of 205 participants. One hundred sixty-two participants (79.0%) were interested in receiving genetic feedback for AUD, with 80.2% of participants who use alcohol indicating interest. The participants indicated most interest in receiving feedback for depression (182 participants; 88.8%) and anxiety (185 participants; 90.2%). A smaller number of participants, yet still a majority, were interested in receiving genetic feedback for NUD (130 participants; 63.4%) and CUD (136 participants; 66.3%). 

Table 2 displays the number of participants that indicated interest in receiving genetic feedback for different combinations of conditions. One hundred twenty-three participants (60.0%) were interested in receiving genetic feedback for all of the five conditions. Only 18 participants (8.8%) were not interested in receiving genetic feedback for any of the conditions.

### 3.2. Variables Related to Interest in Receiving Genetic Feedback

Table 3 presents the descriptives for variables included in the chi-square and *t*-tests used to compare demographic characteristics, substance use and mental health variables, family history, and overall genetic knowledge between those interested in receiving feedback and those not interested in feedback. 

#### 3.2.1. AUD 

Race/ethnicity, cannabis use, and genetic knowledge were associated with interest in receiving genetic feedback for AUD. Individuals who self-identified as White were more likely to indicate interest in receiving genetic feedback for AUD compared to individuals who self-identified as a racial/ethnic minority. Individuals who used cannabis were also more likely to indicate interest in receiving genetic feedback. Finally, overall genetic knowledge was higher in the group of participants interested in receiving genetic feedback for AUD as compared to the group not interested in receiving feedback. None of the mental health or family history variables were significantly associated with interest in receiving feedback for AUD. Results from these analyses are displayed in Table 4.

#### 3.2.2. NUD and CUD 

Both nicotine use and cannabis use were associated with interest in receiving feedback for NUD and CUD, with individuals who reported any nicotine use or cannabis use being more likely to indicate interest in receiving genetic feedback compared to individuals who did not use these substances. Among participants who reported using nicotine, 80% were interested in receiving genetic feedback for NUD and 85% were interested in feedback for CUD. Among participants who reported using cannabis, 74% were interested in feedback for NUD and 87% were interested in receiving feedback for CUD. None of the demographic or family history variables were significantly associated with interest in receiving feedback for either NUD or CUD. Results from these analyses are displayed in Table 4.

#### 3.2.3. Depression and Anxiety 

Higher depressive symptoms, cannabis use, positive family history of alcohol problems and positive family history of depression/anxiety were associated with interest in receiving genetic feedback for depression, while positive family history of depression/anxiety was the only variable associated with indicating interest in receiving genetic feedback for anxiety. Results from these analyses are displayed in Table 5. 

### 3.3. Genetic Knowledge

Overall genetic knowledge surrounding the role of genetic and environmental influences in the development of complex psychiatric conditions was high with the median score being 9 out of 11 items correct (mean = 7.47), but with a wide range (the minimum score was 0 and the maximum score was 11, with an interquartile range of 5 to 10). Notably, 52 participants (25.4%) incorrectly responded to or did not know the answer to more than half of the items, with 20 of these individuals receiving an overall genetic knowledge score of 0. Fifteen participants (7.3%) responded incorrectly to the statement “A person’s substance use disorder is influenced by many different genes” and 49 participants (23.9%) responded that they did not know the correct response to this statement. Table 6 contains the response patterns for all 11 genetic knowledge items. 

None of the variables of interest (e.g., demographic characteristics, substance use and mental health variables, or family history) were associated with overall genetic knowledge. Table 7 displays results from the univariate regression analyses.

## 4. Discussion

To our knowledge, this is the first study to assess interest in receiving genetic feedback for AUD in a sample of young adults. Interest in receiving genetic feedback for AUD, as well as other related substance use disorders and mental health conditions, was high in this sample. These findings align with the previous literature that assesses interest in samples with a broader age range [21,22,31,32,33], and is in keeping with trends of increased access of direct-to-consumer genetic testing [34]. Additionally, our results indicate that interest in receiving genetic feedback for substance use and psychiatric conditions is more general, rather than interest in receiving feedback for only one specific condition, in fact, 60.0% of the sample was interested in receiving feedback for all five conditions. 

Variables related to interest in receiving genetic feedback for AUD include racial/ethnic background, cannabis use, and genetic knowledge. Individuals who self-identified as a racial/ethnic minority were less likely to indicate interest in genetic feedback. This may be related to the historical biomedical abuses related to genetics and racial/ethnic discrimination [35]. However, race/ethnicity was not associated with interest in receiving genetic feedback for the other substance use and psychiatric conditions, suggesting that racial/ethnic differences were specific to interest in receiving genetic feedback for AUD. Since our sample included 18.3% Asian/Asian American and 13.4% African American/Black individuals and lifetime prevalence of alcohol dependence and AUD is lower in these populations [36], this may in part contribute to the racial/ethnic differences in interest in feedback for AUD. Due to the smaller sample size of each racial/ethnic group, we categorized race/ethnicity as binary, which limited our ability to assess differences between each of the subgroups. Additionally, genetic knowledge was only associated with interest in receiving genetic feedback for AUD but not the other conditions. The association between positive family history of alcohol problems and interest in receiving feedback for AUD was marginally significant, which may be a limitation of the smaller sample size. 

Cannabis use was associated with interest in receiving genetic feedback for AUD, as well as NUD, CUD, and depression, suggesting it may be a more general predictor of interest in receiving genetic feedback for substance use and related psychiatric conditions rather than specific to one condition. This may be because cannabis use is a more general indicator of concern about one’s health; these individuals may realize they are engaging in less normative, unhealthy behavior and be concerned. Additionally, both nicotine use and cannabis use were associated with interest in receiving genetic feedback for NUD and CUD, again showing that participants may realize these behaviors are unhealthy, and thus, they are interested in their genetic risk related to developing NUD or CUD. It is also important to note that a majority of participants (53–62%) who do not use nicotine or cannabis were still interested in receiving genetic feedback for NUD and CUD, re-emphasizing the idea that participants have a general curiosity about their genetic risk for substance use conditions. Interestingly, our results indicated that frequency of alcohol use was not associated with interest in receiving genetic feedback for any substance use or psychiatric condition. This may be due to the fact that alcohol use is nearly ubiquitous in our sample, with 91.2% reporting some level of alcohol use.

Our results also showed high interest in receiving feedback for mental health conditions with 90.2% of the sample interested in receiving genetic feedback for anxiety and 88.8% of the sample interested in receiving genetic feedback for depression. Individuals with a positive family history of depression/anxiety were more likely to indicate interest compared to individuals without a family history of depression/anxiety. High interest in these outcomes may reflect the high levels of internalizing symptoms reported in the sample (see Table 3) and more generally in emerging adults [37]. These rising rates of anxiety and depression in this age group is a growing issue that has received a lot of recent attention [38]. In addition, the high rates of interest could be inflated by the higher prevalence of individuals with a positive family history of depression/anxiety in this sample compared to the full sample of individuals invited to complete the survey. Although the only significant association between the variables of interest and interest in receiving feedback for anxiety is family history of depression/anxiety, the results are trending in a similar direction for variables associated with interest in receiving genetic feedback for depression including a positive family history of alcohol problems and higher depressive symptoms. 

It was promising to see that genetic knowledge as it relates to psychiatric conditions is relatively high in this sample. Even though individuals in this sample have at least a partial college education, there is still a sizable proportion (25.4%) of the sample that had a lack of understanding of the role of genetic and environmental factors in complex psychiatric conditions. These findings align with previous literature that demonstrates approximately 25–30% of participants have misconceptions regarding genetics of complex disorders [10,16]. Additionally, demographic characteristics, substance use and mental health variables, and family history were not associated with individuals’ understanding of genetic concepts related to psychiatric conditions. Therefore, additional research is needed to further understand what factors are associated with genetic knowledge. More generally, the fact that a sizable proportion of the sample had a poor understanding of genetic concepts related to substance use and related psychiatric conditions underscores the critical need to provide education alongside receiving genetic feedback for all individuals. Further, because individuals with lower genetic knowledge are less likely to understand complex genetic results, it would be important to examine how best to support their understanding of personalized genetic feedback. 

Our findings need to be interpreted in light of several limitations. First, one of the inclusion criteria for participant recruitment was having available GWAS data. This may contribute to the high interest because these participants already consented and provided DNA samples and could be more open to receiving genetic feedback compared to the general population. However, it is important to note that nearly all of the individuals who participated in the overall S4S registry survey component provided a DNA sample (97%), suggesting they are not a highly selected sample. Second, in regards to genetic knowledge, this sample is educated, and have received at least a partial college education, which could inflate the genetic knowledge scores. A more diverse sample is needed to understand genetic knowledge as it relates to psychiatric conditions in the general public. Third, our sample was primarily female (76.5%) and White (58.9%), thus limiting our ability to test sex differences and differences between racial/ethnic background. Our findings may be more representative for females. 

Despite these limitations, the findings of this study elucidate the importance of additional research related to the return of genetic feedback for AUD and related outcomes. Individuals are highly interested in receiving their genetic feedback for substance use disorders and mental health conditions, but there is a sizable proportion of individuals who might not have the knowledge needed to properly interpret and understand their results. Research is needed to develop and evaluate effective and efficient ways to communicate complex personalized genetic risk information for AUD and related substance use and psychiatric conditions, as well as to understand the impact of receiving genetic risk information for these conditions on the individual. 

## 5. Conclusions

Interest in receiving genetic feedback for AUD, as well as other related substance use disorders and mental health outcomes, is high. However, there is a lack of understanding about the role of genetic influences in the development of substance use disorders and related mental health outcomes in a sizable minority of individuals. Therefore, additional research is needed to design and evaluate the effectiveness of educational materials that address the lack of knowledge before genetic feedback for AUD and related outcomes can be returned in a way that benefits the individuals receiving them.

## Figures and Tables

**Table 1:** Frequencies of interest in receiving genetic feedback for the five substance use and psychiatric conditions in the full sample.

Outcome	Interested (%)	Not Interested (%)
Alcohol use disorder	79.0%	21.0%
Nicotine use disorder	63.4%	36.6%
Cannabis use disorder	66.3%	33.7%
Depression	88.8%	11.2%
Anxiety	90.2%	9.8%

Note: Sample size = 205 participants.

**Table 2:** Patterns of interest in receiving genetic feedback across the five substance use and psychiatric conditions.

	Number of Participants	Frequency
All 5 conditions	123	60.0%
AUD, Depression, Anxiety	23	11.2%
None of the conditions	18	8.8%
Depression & Anxiety only	16	7.8%
All conditions except NUD	8	3.9%
All conditions except CUD	6	2.9%
Only Anxiety	4	2.0%
CUD, Depression, Anxiety	3	1.5%
AUD & Anxiety only	1	0.5%
All conditions except AUD	1	0.5%
AUD & Depression only	1	0.5%
CUD & Depression only	1	0.5%

Note: Sample size = 205 participants. AUD (Alcohol Use Disorder), NUD (Nicotine Use Disorder), CUD (Cannabis Use Disorder).

**Table 3:** Descriptives of study variables used to examine the relationship between demographic characteristics, substance use and mental health variables, and family history and individuals’ interest in receiving genetic feedback.

Variables	Mean (*SD*)	*n* (%)
Age	24.48 (*0.36*)	-
Sex ^a^	-	156 (76.5%)
Race/Ethnicity ^a^	-	83 (41.1%)
Depressive Symptoms	11.37 (*3.95*)	-
Anxiety Symptoms	8.8 (*3.8*)	-
Alcohol Frequency	2.9 (*1.08*)	-
Nicotine Use ^a^	-	40 (19.5%)
Cannabis Use ^a^	-	82 (40.4%)
Family History of Alcohol Problems ^a^	-	120 (59.4%)
Family History of Depression/Anxiety ^a^	-	143 (71.1%)
Family History of Other Drug Problems ^a^	-	90 (44.6%)
Overall Genetic Knowledge	7.47 (*3.58*)	-

Note: ^a^ For binary variables, *n* and proportion for response category = 1 were reported. Sex is coded 1 = female, 0 = male; Race/ethnicity is coded 1 = racial/ethnic minority, 0 = White; Nicotine use is coded 1 = nicotine use in the past 30 days, 0 = no nicotine use in the past 30 days; Cannabis use is coded 1 = cannabis use, 0 = no cannabis use; Family history of alcohol problems is coded 1 = positive family history of alcohol problems, 0 = no family history of alcohol problems; Family history of depression/anxiety is coded 1 = positive family history of depression/anxiety, 0 = no family history of depression/anxiety; Family history of other drug problems is coded 1 = positive family history of other drug problems, 0 = no family history of other drug problems.

**Table 4:** Results of the chi-square and *t*-tests to compare demographic characteristics, substance use and mental health variables, family history, and overall genetic knowledge between those interested in receiving feedback for alcohol use disorder, nicotine use disorder, and cannabis use disorder and those not interested.

	Alcohol Use Disorder			Nicotine Use Disorder			Cannabis Use Disorder		
Variable	Interested (*n* = 162)	Not Interested (*n* = 43)	t/χ^2^ (*p* Value)	Interested (*n* = 130)	Not Interested (*n* = 75)	t/χ^2^ (*p* Value)	Interested (*n* = 136)	Not Interested (*n* = 69)	t/χ^2^ (*p* Value)
Age ^a^	24.49 (0.35)	24.43 (0.38)	0.892 (0.376)	24.5 (0.36)	24.43 (0.35)	1.281 (0.202)	24.51 (0.35)	24.42 (0.36)	1.672 (0.097)
Sex									
Male	77%	23%	0.128 (0.721)	58%	42%	0.649 (0.421)	67%	33%	0.007 (0.935)
Female	79%	21%		65%	35%		66%	34%	
Race/Ethnicity									
White	**84%**	**16%**	**4.893 (0.027)**	66%	34%	0.888 (0.346)	67%	33%	0.103 (0.749)
Rac/Eth Minority	**71%**	**29%**		59%	41%		65%	35%	
Depressive Symptoms ^a^	11.48 (3.83)	10.93 (4.43)	0.740 (0.462)	11.55 (3.71)	11.05 (4.36)	0.817 (0.415)	11.57 (3.83)	10.97 (4.20)	0.984 (0.327)
Anxiety Symptoms ^a^	8.75 (3.71)	9.00 (4.17)	−0.358 (0.721)	8.65 (3.51)	9.05 (4.28)	−0.684 (0.495)	8.81 (3.70)	8.78 (4.03)	0.051 (0.960)
Alcohol Frequency ^a^	1.93 (1.05)	1.77 (1.19)	0.825 (0.413)	1.88 (1.09)	1.93 (1.08)	−0.359 (0.720)	1.91 (1.06)	1.87 (1.12)	0.259 (0.796)
Nicotine Use									
No	78%	22%	1.071 (0.301)	**59%**	**41%**	**5.892 (0.015)**	**62%**	**38%**	**7.748 (0.005)**
Yes	85%	15%		**80%**	**20%**		**85%**	**15%**	
Cannabis Use									
No	**74%**	**26%**	**4.437 (0.035)**	**56%**	**44%**	**6.983 (0.008)**	**53%**	**47%**	**24.907 (0.000)**
Yes	**87%**	**13%**		**74%**	**26%**		**87%**	**13%**	
FH of ALC Prob									
No	72%	28%	3.766 (0.052)	63%	37%	0.012 (0.913)	67%	33%	0.004 (0.952)
Yes	83%	17%		64%	36%		67%	33%	
FH of Dep/Anx									
No	74%	26%	0.968 (0.325)	60%	40%	0.392 (0.531)	57%	43%	3.502 (0.061)
Yes	80%	20%		65%	35%		71%	29%	
FH of Drug Prob									
No	80%	20%	0.406 (0.524)	65%	35%	0.189 (0.664)	68%	32%	0.119 (0.730)
Yes	77%	23%		62%	38%		66%	34%	
Genetic Knowledge ^a^	**7.77 (3.54)**	**6.35 (3.57)**	**2.327 (0.023)**	7.65 (3.66)	7.17 (3.45)	0.924 (0.357)	7.75 (3.59)	6.93 (3.53)	1.568 (0.119)

Note: ^a^ denotes variables that are quantitative. % are reported for categorical variables and mean (SD) are presented for continuous. Boldface indicates estimate *p* < 0.05. Rac/Eth Minority = Racial/Ethnic Minority. FH = Family History. ALC Prob = Alcohol Problems. Dep = Depression. Anx = Anxiety. Drug Prob = Other Drug Problems. Total *N* = 205.

**Table 5:** Results of the chi-square and *t*-tests to compare demographic characteristics, substance use and mental health variables, family history, and overall genetic knowledge between those interested in receiving feedback for depression and anxiety and those not interested.

	Depression			Anxiety		
Variable	Interested (*n* = 182)	Not Interested (*n* = 23)	t/χ^2^ (*p* Value)	Interested (*n* = 185)	Not Interested (*n* = 20)	t/χ^2^ (*p* Value)
Age ^a^	24.47 (0.35)	24.51 (0.45)	−0.393 (0.698)	24.48 (0.35)	24.48 (0.44)	0.025 (0.980)
Sex						
Male	90%	10%	0.046 (0.830)	90%	10%	0.027 (0.870)
Female	88%	12%		90%	10%	
Race/Ethnicity						
White	90%	10%	0.487 (0.485)	92%	8%	1.775 (0.183)
Rac/Eth Minority	87%	13%		87%	13%	
Depressive Symptoms ^a^	**11.67 (3.91)**	**8.86 (3.44)**	**3.558 (0.001)**	11.55 (3.91)	9.58 (4.02)	2.043 (0.053)
Anxiety Symptoms ^a^	8.91 (3.80)	7.86 (3.78)	1.227 (0.231)	8.79 (3.76)	8.84 (4.26)	−0.047 (0.963)
Alcohol Frequency ^a^	1.87 (1.07)	2.09 (1.16)	−0.835 (0.411)	1.87 (1.09)	2.15 (1.04)	−1.138 (0.267)
Nicotine Use						
No	88%	12%	0.690 (0.406)	89%	11%	1.277 (0.259)
Yes	93%	7%		95%	5%	
Cannabis Use						
No	**85%**	**15%**	**5.056 (0.025)**	88%	12%	1.726 (0.189)
Yes	**95%**	**5%**		94%	6%	
FH of ALC Prob						
No	**83%**	**17%**	**4.425 (0.035)**	85%	15%	3.467 (0.063)
Yes	**93%**	**7%**		93%	7%	
FH of Dep/Anx						
No	**79%**	**21%**	**6.879 (0.009)**	**83%**	**17%**	**4.837 (0.028)**
Yes	**92%**	**8%**		**93%**	**7%**	
FH of Drug Prob						
No	88%	12%	0.309 (0.578)	88%	12%	0.820 (0.365)
Yes	90%	10%		92%	8%	
Genetic Knowledge ^a^	7.57 (3.59)	6.7 (3.55)	1.114 (0.275)	7.57 (3.56)	6.55 (3.75)	1.165 (0.256)

Note: ^a^ denotes variables that are quantitative. % are reported for categorical variables and mean (SD) are presented for continuous. Boldface indicates estimate *p* < 0.05. Rac/Eth Minority = Racial/Ethnic Minority. FH = Family History. ALC Prob = Alcohol Problems. Dep = Depression. Anx = Anxiety. Drug Prob = Other Drug Problems. Total *N* = 205.

**Table 6:** Response patterns for the 11 genetic knowledge items.

Statement	Correct	Incorrect	Don’t Know
A gene codes directly for a psychiatric condition. *(false)*	42.4%	21.0%	36.6%
Most psychiatric conditions are caused by a single gene. (*false)*	61.0%	6.3%	32.7%
A single gene can influence several different psychiatric conditions. *(true)*	71.2%	7.3%	21.5%
A person’s substance use disorder is influenced by one gene only. *(false)*	74.1%	2.4%	23.4%
A person’s depression is influenced by one gene only. *(false)*	77.1%	1.5%	21.5%
Most psychiatric conditions are influenced by many different genes. *(true)*	73.7%	2.9%	23.4%
Most psychiatric conditions are caused by environmental factors only (such as parenting or trauma). *(false)*	58.5%	19.5%	22.0%
A gene can only influence a single psychiatric condition. *(false)*	72.2%	3.9%	23.9%
Most psychiatric conditions are caused by both genes and environmental factors. *(true)*	80.0%	5.9%	14.1%
A person’s substance use disorder is influenced by many different genes. *(true)*	68.8%	7.3%	23.9%
A person’s depression is influenced by many different genes. *(true)*	68.3%	7.3%	24.4%

Note: Sample size = 205 participants.

**Table 7:** Results from the univariate regression analyses assessing associations between demographic characteristics, substance use and mental health variables, and family history and overall genetic knowledge.

	Genetic Knowledge			
	Univariate Analyses			
Variable	Estimate	Std. Error	z Value	*p* Value
Age	1.374	0.746	1.842	0.067
Sex	0.085	0.594	0.143	0.886
Race/Ethnicity	−0.779	0.499	−1.561	0.120
Depressive Symptoms	−0.013	0.063	−0.206	0.837
Anxiety Symptoms	−0.042	0.066	−0.644	0.520
Alcohol Frequency	−0.310	0.231	−1.340	0.182
Nicotine Use (Binary)	−0.681	0.631	−1.079	0.282
Cannabis Use (Binary)	−0.397	0.509	−0.780	0.436
Family History of Alcohol Problems	0.145	0.509	0.285	0.776
Family History of Depression/Anxiety	0.548	0.553	0.991	0.323
Family History of Other Drug Problems	−0.630	0.501	−1.257	0.210

Note: Sex is coded 1 = female, 0 = male; Race/ethnicity is coded 1 = racial/ethnic minority, 0 = White; Nicotine use is coded 1 = nicotine use in the past 30 days, 0 = no nicotine use in the past 30 days; Cannabis use is coded 1 = cannabis use, 0 = no cannabis use; Family history of alcohol problems is coded 1 = positive family history of alcohol problems, 0 = no family history of alcohol problems; Family history of depression/anxiety is coded 1 = positive family history of depression/anxiety, 0 = no family history of depression/anxiety; Family history of other drug problems is coded 1 = positive family history of other drug problems, 0 = no family history of other drug problems.

## Supplementary Materials

The following are available online at https://www.mdpi.com/2076-3425/10/12/1007/s1, survey items.
