# Supplementary material for: Interest in Genetic Feedback for Alcohol Use Disorder and Related Substance Use and Psychiatric Outcomes among Young Adults
Source: Brain Sci. 2020 Dec 18;10(12):1007. doi: 10.3390/brainsci10121007 (PMC7766419; doi:10.3390/brainsci10121007)
Supplement: Supplementary file 1 [file brainsci-10-01007-s001.pdf]

Supplementary Materials

A. Survey Items

DEMOGRAPHIC INFORMATION

1. What is your current age?

[ ]

2. What sex were you assigned at birth, meaning on your original birth certificate?

( ) Male
( ) Female
( ) I choose not to answer

3. Which one of these groups best describes you? (please choose only one)

( ) American Indian/Alaska Native
( ) Asian
( ) Black/African American
( ) Hispanic/Latino
( ) More than one race
( ) Native Hawaiian/Other Pacific Islander
( ) Unknown
( ) White
( ) I choose not to answer

INTEREST IN GENETIC FEEDBACK

4. Would you be interested in receiving personalized genetic feedback for any of the following mental health conditions? Select all that apply.

( ) Alcohol use disorder
( ) Nicotine use disorder
( ) Cannabis use disorder
( ) Depression
( ) Anxiety
( ) Other substance use or behavioral outcomes [ ]
( ) None of the above
( ) Unsure
( ) I choose not to answer

GENETIC KNOWLEDGE

5. Read each statement below and select one box (True, False or Don´t know). Please only choose "don't know" if you do not understand the statement. *Items adapted from Carver et al. 2017.*

Q.		True	False	Don't know	I choose not to answer
a	A gene codes directly for a psychiatric condition.				
b	Most psychiatric conditions are caused by a single gene.				
c	A single gene can influence several different psychiatric conditions.				
d	A person's substance use disorder is influenced by one gene only.				
e	A person's depression is influenced by one gene only.				
f	Most psychiatric conditions are influenced by many different genes.				
g	Most psychiatric conditions are caused by environmental factors only (such as parenting or trauma).				
h	A gene can only influence a single psychiatric condition.				
i	Most psychiatric conditions are caused by both genes and environmental factors.				

j	A person's substance use disorder is influenced by many different genes.				
k	A person's depression is influenced by many different genes.				

SUBSTANCE USE

6. How often do you have a drink containing alcohol? *Item from AUDIT (Bohn et al., 1995).*

( ) Never
( ) Monthly or less
( ) 2 to 4 times a month
( ) 2 to 3 times a week
( ) 4 or more times a week
( ) I choose not to answer

7. How frequently did you use a nicotine product (cigarettes, e-cigarettes, hookah, cigars, dip, etc) <u>in the last 30 days</u>? *Item adapted from the National Survey on Drug Use and Health (SAMSHA, 2014).*

( ) Never in the last month
( ) Once or twice
( ) A few days (3 to 4 days a month)
( ) A couple of days a week (5 to 11 days a month)
( ) Three times a week (12 to 14 days a month)
( ) Most days of the week (15 to 25 days a month)
( ) Daily or almost daily (26 to 30 days a month)
( ) I choose not to answer

8. How often do you use cannabis (marijuana, hashish, THC, ganja, etc)? *Item from CUDIT (Adamson & Sellman, 2003).*

( ) Never
( ) Monthly or less
( ) 2 to 4 times a month
( ) 2 to 3 times a week
( ) 4 or more times a week
( ) I choose not to answer

<u>ANXIETY AND DEPRESSIVE SYMPTOMS</u>

9. The next questions ask about some problems and feelings which people sometimes have. Please give the answer which best describes how much discomfort that problem has caused you during the last 30 days, including today. *Items adapted from the Symptom Checklist-90 (Derogatis & Cleary, 1977).*

a. nervousness or shakiness inside

( ) Not at all
( ) A little bit
( ) Moderately
( ) Quite a bit
( ) Extremely
( ) I choose not to answer

b. suddenly scared for no reason

( ) Not at all
( ) A little bit
( ) Moderately
( ) Quite a bit
( ) Extremely
( ) I choose not to answer

c. feeling blue

( ) Not at all
( ) A little bit
( ) Moderately
( ) Quite a bit
( ) Extremely
( ) I choose not to answer

d. worrying too much about things

( ) Not at all
( ) A little bit
( ) Moderately
( ) Quite a bit
( ) Extremely
( ) I choose not to answer

e. feeling no interest in things

( ) Not at all
( ) A little bit
( ) Moderately
( ) Quite a bit
( ) Extremely
( ) I choose not to answer

f. feeling fearful

- ( ) Not at all
- ( ) A little bit
- ( ) Moderately
- ( ) Quite a bit
- ( ) Extremely
- ( ) I choose not to answer

g. feeling hopeless about the future

- ( ) Not at all
- ( ) A little bit
- ( ) Moderately
- ( ) Quite a bit
- ( ) Extremely
- ( ) I choose not to answer

h. spells of terror or panic

- ( ) Not at all
- ( ) A little bit
- ( ) Moderately
- ( ) Quite a bit
- ( ) Extremely
- ( ) I choose not to answer

FAMILY HISTORY

Items from Kendler et al. 2015.

10. Do you think your biological mother has ever had a drinking problem? (By drinking problem we mean that her drinking caused problems at home, at work, with her health, or with the police, or that she received alcohol treatment.)

- ( ) Yes
- ( ) No
- ( ) I don't know
- ( ) I choose not to answer

11. Do you think your biological mother has ever had problems with other drugs? (By problems with drugs we mean that her drug use caused problems at home, at work, with her health, or with the police, or that she received drug treatment.)

- ( ) Yes
- ( ) No
- ( ) I don't know
- ( ) I choose not to answer

12. Do you think your biological mother has ever had problems with depression or anxiety?

- ( ) Yes
- ( ) No
- ( ) I don't know

( ) I choose not to answer

13. Do you think your biological father has ever had a drinking problem?

( ) Yes
( ) No
( ) I don't know
( ) I choose not to answer

14. Do you think your biological father has ever had problems with other drugs?

( ) Yes
( ) No
( ) I don't know
( ) I choose not to answer

15. Do you think your biological father has ever had problems with depression or anxiety?

( ) Yes
( ) No
( ) I don't know
( ) I choose not to answer

16. Do you think your aunts, uncles, or grandparents (counting only those who are blood relations of your parents) have ever had a drinking problem?

( ) Yes
( ) No
( ) I don't know
( ) I choose not to answer

17. Do you think your biological aunts, uncles, or grandparents have ever had problems with other drugs?

( ) Yes
( ) No
( ) I don't know
( ) I choose not to answer

18. Do you think your biological aunts, uncles, or grandparents have ever had problems with depression or anxiety?

( ) Yes
( ) No
( ) I don't know
( ) I choose not to answer

19. Do you think your biological siblings (same biological mother and father) have ever had a drinking problem?

( ) Yes
( ) No
( ) I don't know
( ) I choose not to answer

20. Do you think your biological siblings have ever had problems with other drugs?

( ) Yes
( ) No
( ) I don’t know
( ) I choose not to answer

21. Do you think your biological siblings have ever had problems with depression or anxiety?

( ) Yes
( ) No
( ) I don’t know
( ) I choose not to answer
